# Structure-Based De Novo Design for the Discovery of Miniprotein Inhibitors Targeting Oncogenic Mutant BRAF

**DOI:** 10.3390/ijms25105535

**Published:** 2024-05-19

**Authors:** Jae Min Ham, Myeongbin Kim, Taeho Kim, Seong Eon Ryu, Hwangseo Park

**Affiliations:** 1 Department of Bioengineering, College of Engineering, Hanyang University, 222 Wangsimri-ro, Seong-dong-gu, Seoul 04763, Republic of Korea; hjm5588@hanyang.ac.kr (J.M.H.); clearwds@naver.com (M.K.); 2 Department of Bioscience and Biotechnology, Sejong University, 209 Neungdong-ro, Kwangjin-gu, Seoul 05006, Republic of Korea; taehok88@gmail.com

**Keywords:** oncogenic mutant BRAF, miniprotein inhibitor, de novo design, anticancer agent

## Abstract

Being a component of the Ras/Raf/MEK/ERK signaling pathway crucial for cellular responses, the VRAF murine sarcoma viral oncogene homologue B1 (BRAF) kinase has emerged as a promising target for anticancer drug discovery due to oncogenic mutations that lead to pathway hyperactivation. Despite the discovery of several small-molecule BRAF kinase inhibitors targeting oncogenic mutants, their clinical utility has been limited by challenges such as off-target effects and suboptimal pharmacological properties. This study focuses on identifying miniprotein inhibitors for the oncogenic V600E mutant BRAF, leveraging their potential as versatile drug candidates. Using a structure-based de novo design approach based on binding affinity to V600E mutant BRAF and hydration energy, 39 candidate miniprotein inhibitors comprising three helices and 69 amino acids were generated from the substructure of the endogenous ligand protein (14-3-3). Through in vitro binding and kinase inhibition assays, two miniproteins (63 and 76) were discovered as novel inhibitors of V600E mutant BRAF with low-micromolar activity, with miniprotein 76 demonstrating a specific impediment to MEK1 phosphorylation in mammalian cells. These findings highlight miniprotein 76 as a potential lead compound for developing new cancer therapeutics, and the structural features contributing to its biochemical potency against V600E mutant BRAF are discussed in detail.

## 1. Introduction

VRAF murine sarcoma viral oncogene homologue B1 (BRAF) is a serine/threonine-specific protein kinase, and it belongs to the RAF kinase family (ARAF, BRAF, and CRAF). Activation of the RAS/RAF/MEK/ERK signaling pathway induces cell growth, division, and differentiation in normal cells. However, hyperactivation of this signaling pathway causes excessive cell proliferation and culminates in the pathogenesis of human cancers, which has been exemplified by various oncogenic mutations in RAS and BRAF genes [1,2,3]. For example, the activating mutations in the BRAF gene accounts for 40–60% of melanomas [4]. Most oncogenic BRAF mutations take place in the kinase domain. Among them, a single mutation at residue 600 from Val to Glu accounts for approximately 90% of BRAF mutants [5]. V600E mutant BRAF activates downstream signal transduction in a constitutive fashion and thereby facilitates the initiation and progression of tumor cells in a variety of human cancers. It is also known that V600E mutant BRAF plays a critical role in maintaining the functions of tumor cells [6]. Accordingly, the inhibition of V600E mutant BRAF is considered a promising strategy for the development of new cancer medicines.

In accordance with the pharmaceutical interest, three-dimensional (3D) structures of BRAF kinase domains of both the wild type and the V600E mutant have been reported in complexes with various small-molecule inhibitors [7,8]. The abundance of structural information on the interactions between BRAF and inhibitors has promoted the design and discovery of a variety of ATP-competitive inhibitors. Indeed, a great deal of scientific endeavors have been devoted to identifying potent BRAF inhibitors that may develop into a new anticancer medicine. First-generation BRAF inhibitors, including SB-590885 and PLX-4720, exhibit low biochemical potency against the oncogenic mutant as well as poor specificity in the inhibition of BRAF [9]. This has motivated the discovery of second-generation inhibitors with improved biochemical potency against V600E mutant BRAF. Although it has been shown in several preclinical and clinical studies that second-generation inhibitors can suppress the progression of tumors containing V600E mutant BRAF, some of them have the effect of activating BRAF activity through downstream ERK signaling [10]. This paradoxical activation may lead to the pathogenesis of new cancers in normal cells [11]. In addition, the discovery of third-generation inhibitors has aimed at preventing the dimerization of BRAF to overcome the resistance of the associated cancer cells to first- and second-generation inhibitors [12]. Because the application of third-generation inhibitors has also been limited by paradoxical activation, some new design strategies have been suggested, including the dual inhibition of V600E mutant BRAF and MEK, as well as allosteric inhibition to avoid BRAF dimerization [13]. Unexpected off-target activities have thus made it difficult for small-molecule BRAF inhibitors to develop into a new cancer medicine. Nonetheless, there has been an active pursuit in discovering BRAF inhibitors because of advances in combination therapy and new design methods aimed at addressing the selectivity issue [13].

Recently, miniproteins comprising less than 100 amino acids have drawn much interest as a new therapeutic resource because they are capable of inhibiting the target protein specifically by binding to the interface of protein–protein interactions instead of a small hydrophobic pocket in the active site [14]. Due to the stability of 3D structures and the relatively good permeability across the cell membrane, miniproteins are also meritorious over antibodies in achieving biochemical potency against intracellular targets. With respect to the availability of general development methods, structure-based de novo design has proved useful for identifying the potent miniprotein inhibitors of various target proteins [15,16,17].

In the present study, we aimed to find the miniprotein inhibitors of V600E mutant BRAF by combining structure-based de novo design and subsequent in vitro binding and kinase inhibition assays. These miniprotein inhibitors were further evaluated using mammalian cells to confirm the presence of anticancer activity. It was shown that miniprotein BRAF inhibitors with anticancer activity could be identified using conventional computer-aided molecular design as efficiently as small-molecule inhibitors with similar efficacy.

## 2. Results and Discussion

### 2.1. De Novo Design of Candidate Miniprotein Inhibitors of V600E Mutant BRAF^15mut^

**Figure 1:**
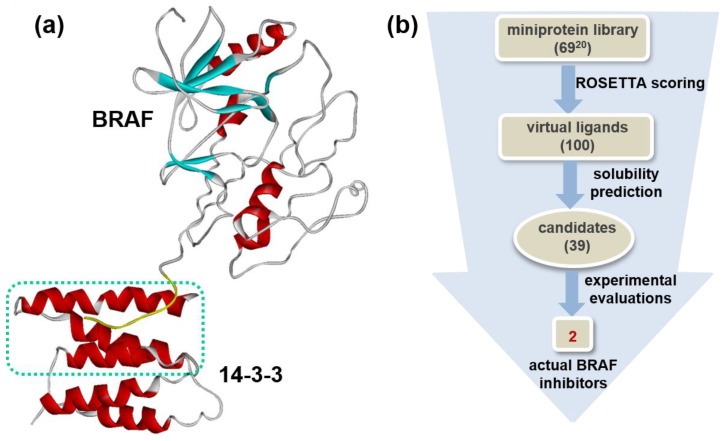
(**a**) X-ray structure of BRAF in complex with 14-3-3 (PDB entry: 4MNE). Indicated in green dotted box is the three α helices (residues 164–232) used as a structural motif of miniprotein ligands. (**b**) Flowchart for the discovery of miniprotein inhibitors of V600E mutant BRAF via de novo design and experimental validations.

In general, it is more difficult to design the miniprotein binders than the small-molecule ligands because the structural stability of a protein fold should be taken into account, as well as the binding affinity to the target protein. For example, only 0.2 to 3% of candidate miniprotein binders derived from the conventional protein design methods were found to exhibit the biochemical potency in experimental validations [17]. To enhance the possibility of finding the actual miniprotein inhibitors, the structure-based de novo design was carried out in this work under consideration of the two points in addition to the binding affinity to V600E mutant BRAF^15mut^. First, the substructure of an endogenous ligand protein (14-3-3) was used as the starting structure instead of constructing the new structural scaffolds. As shown in Figure 1a, this structural motif comprised three α helices (residues 164–232) bound to the C-terminal tail of BRAF. The use of a natural protein as a starting point seemed to be effective in the rapid identification of high-affinity miniprotein binders by reducing the false positives as exemplified in designing the inhibitors of angiotensin-converting enzyme 2 [16]. Second, the candidate miniproteins selected using the calculated binding affinity to V600E mutant BRAF^15mut^ were further screened according to the aqueous solubility to exclude the insoluble and poorly soluble miniproteins in the early stage of discovery. This step seemed to be necessary because miniproteins comprising several helices tend to be hydrophobic due to the abundance of nonpolar residues such as Val and Leu at the interface between helices [18].

**Figure 2:**
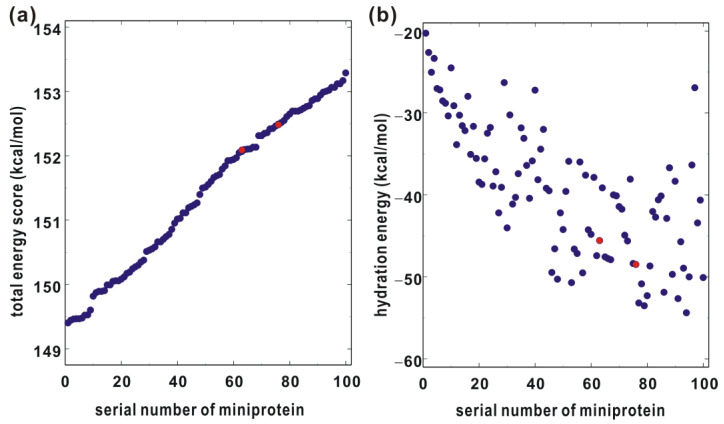
(**a**) Total energy scores of 100 miniproteins with respect to binding to V600E mutant BRAF^15mut^ and (**b**) their calculated hydration free energies. The positions of miniproteins 63 and 76 are indicated in red.

Starting from the binding configuration derived from the protein–protein docking simulations, the structure-based de novo design of miniproteins was carried out in order to optimize the structural stability and the binding affinity to V600E mutant BRAF^15mut^ in a simultaneous fashion. After selecting 100 top-scoring candidates out of 1000 miniproteins generated with the ROSETTA scoring function, the second screening was conducted to exclude potentially insoluble miniproteins as illustrated in Figure 1b. More specifically, the hydration free energy of each candidate miniprotein was calculated using the potential energy function, the atomic volume (*V_j_*), maximum atomic occupancy (*O_i_^max^*), and atomic solvation parameters (*S_i_*), which were optimized with the hydration free energy data of dipeptides [19]. Figure 2 shows the total binding energy scores of the initial candidates in comparison to their calculated hydration energies. The hydration energies ranged from −55 to −20 kcal/mol (Figure 2b) with no correlation with the total energy score for binding to V600E mutant BRAF^15mut^ (Figure 2a). Since the variations in the calculated binding affinities of miniproteins appeared to be negligible when compared to those in the hydration energies, the initial 100 virtual hits selected in the reverse order of the former were rescored according to the latter. The final virtual hits included only 39 miniproteins with a hydration energy lower than −42 kcal/mol. This step seemed to be necessary for finding the actual miniprotein BRAF inhibitors because the protein solubility is the most important prerequisite for structural and biochemical studies [20]. Subsequently, all 39 candidate miniprotein binders of V600E mutant BRAF^15mut^ were prepared for the experimental evaluation of inhibitory activity.

### 2.2. Expression and Purification of Candidate Miniprotein Inhibitors

**Figure 3:**
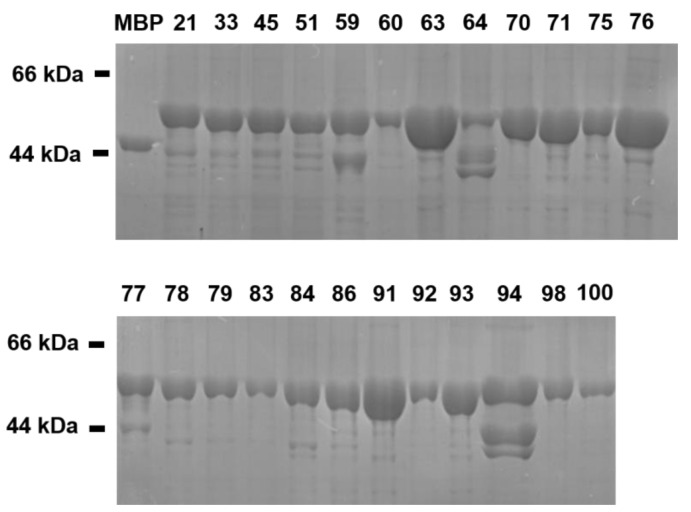
Coomassie-blue-stained SDS-PAGE images of the purified MBP and the MBP-tagged candidate miniprotein inhibitors. Miniproteins expressed using the *E. coli* expression system were purified with affinity chromatography. The number at the top of each lane indicates the serial number of a miniprotein.

All 39 candidate miniproteins designed according to the binding affinity to V600E mutant BRAF^15mut^ and aqueous solubility were expressed using the *E. coli* strain BL21 (DE3), in which DNA encoding each miniprotein was transformed. As a consequence, 24 of the 39 candidate miniprotein inhibitors were synthesized successfully via bacterial expressions. All the purified miniproteins were detected using the SDS-PAGE method, the results of which are shown in Figure 3 along with those for MBP with Myc-tag at the C-terminus. Most miniproteins were obtained in water-soluble form, although several of them including miniprotein 64 and 94 revealed multiple bands due most probably to degradation by low thermal stability.

Table 1 lists the amino acid sequences of 24 miniproteins prepared for biochemical evaluations of the inhibitory activity against V600E mutant BRAF^15mut^. When the amino acid sequences of the miniproteins comprising three α-helices were analyzed, it followed immediately that the ratios of hydrophobic residues and cysteine fell within 60% and 4.35%, respectively. This is consistent with the requirements of low populations of hydrophobic residues and cysteine for aqueous solubility [21,22].

### 2.3. Expression and Purification of BRAF Kinase Domain and MEK1

To prepare a proper in vitro assay system for measuring the kinase activity of V600E mutant BRAF^15mut^, the target of phosphorylation (MEK1) was also produced using the *E. coli* expression system. Because the wild-type MEK1 might be auto-phosphorylated via dimerization [23], a kinase-dead version of MEK1 involving the mutation of Lys at residue 97 to Met was prepared instead of the wild type. Although the kinase domain of wild-type BRAF is known to be expressed well only in *Spodoptera frugiperda* insect cells [24], the *E. coli* expression system could also be applicable by introducing 16 solubilizing mutations [25]. Despite such multiple mutations, the kinase activity of BRAF turned out to be conserved well because all the mutations are located in the C-terminal lobe and reside far from the active site. Therefore, the *E. coli* expression system was used in this work for preparing the kinase domain of BRAF as well as MEK1 instead of insect cells because the former is more productive and technically feasible than the latter. Among a total of 16 mutational residues, Phe667 was kept intact to produce the variant of BRAF containing 15 mutations (BRAF^15mut^). This was based on the previous experimental finding that Phe667 played an important role in binding to MEK1 and phosphorylation [26]. In addition to the solubilizing mutations, Val at residue 600 of BRAF^15mut^ was further mutated to Glu to generate a proper model system for the constitutively active V600E mutant BRAF [25,27]. The validity of the recombinant protein as a model system for V600E mutant BRAF was further confirmed by its kinase activity with respect to MEK1 as detailed below.

**Figure 4:**
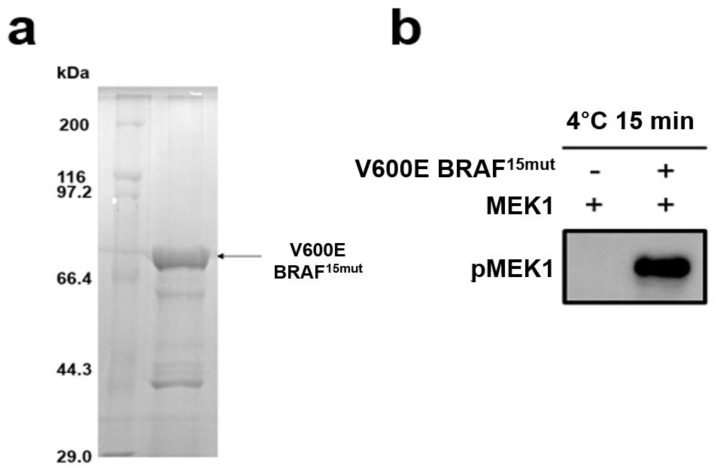
(**a**) Coomassie-blue-stained SDS-PAGE gel image of purified V600E mutant BRAF^15mut^. (**b**) Western blot results for in vitro kinase activity of V600E mutant BRAF^15mut^ with respect to MEK1.

Figure 4 shows the Coomassie-blue-stained SDS-PAGE gel image of the V600E mutant BRAF^15mut^ produced with the *E. coli* expression system, along with the results of in vitro kinase assays with MEK1. V600E mutant BRAF^15mut^ appears to be expressed in the soluble form with the dominant band between 66 and 97 kDa markers (Figure 4a). In vitro kinase activity of the purified V600E mutant BRAF^15mut^ could also be confirmed with the Western blot of the mixture containing MEK1, V600E mutant BRAF^15mut^, and anti-pMEK1 antibody. The results show that MEK1 is phosphorylated only in the presence of V600E mutant BRAF^15mut^ (Figure 4b), implying the conservation of kinase activity in V600E mutant BRAF^15mut^. In addition, the lack of phosphorylated MEK1 in the absence of V600E mutant BRAF^15mut^ indicates that the auto-phosphorylation function of MEK1 was negated successfully via introduction of the K97M mutation. On the basis of the reasonably good kinase activity and the lack of problematic auto-phosphorylation, V600E mutant BRAF^15mut^ and K97M mutant MEK1 were used to measure the biochemical potencies of 24 candidate miniprotein inhibitors.

### 2.4. BRAF-MEK1 Binding Assays

**Figure 5:**
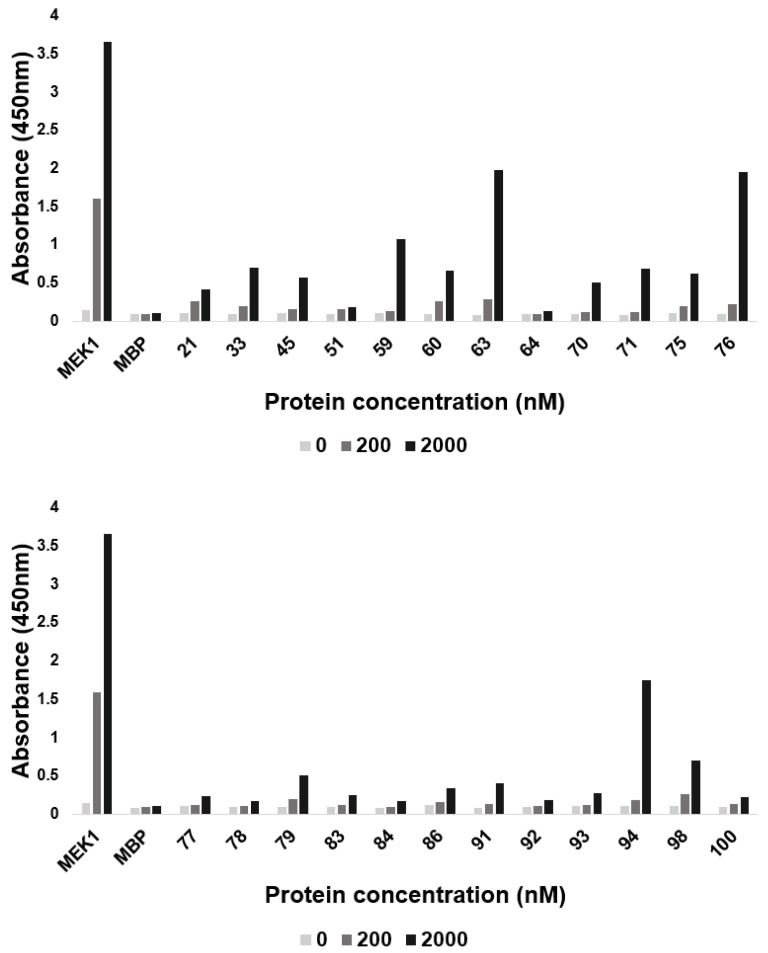
The absorbances of 24 candidate miniproteins in the presence of V600E mutant BRAF^15mut^, in comparison to those of MEK1 and MBP.

The binding affinities of 24 candidate miniprotein inhibitors with respect to V600E mutant BRAF^15mut^ were investigated using the ELISA method. MEK1 and MBP served as the positive and negative controls in the binding assays, respectively. As summarized in Figure 5, three miniproteins (63, 76, and 94) of 24 candidates exhibited more than half of the binding affinity of MEK1 at 2 μM. This is remarkable because the number of amino acids of miniproteins is 3.2-fold less than that of MEK1. A sharp increase in the absorbance with the increase in miniprotein concentration confirmed that miniproteins 63, 76, and 94 would be the actual binders of V600E mutant BRAF^15mut^. Nonetheless, miniprotein 94 was excluded in further investigations because it remained unstable after purification and culminated in degradation (Figure 3). Structural and amino-acid sequence alignments of miniproteins 63 and 76 with respect to 14-3-3 are provided in Supplementary Material (Figures S1 and S2).

### 2.5. In Vitro Kinase Inhibition Assays

**Figure 6:**
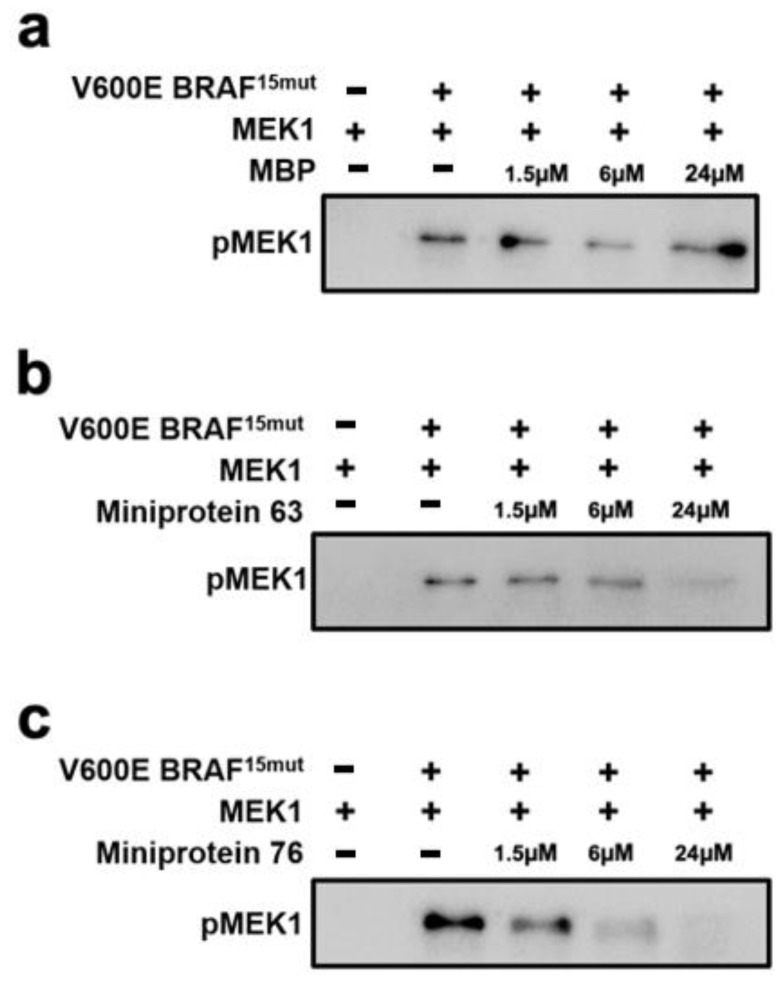
Western blot analysis of in vitro kinase assay results in the presence of (**a**) MBP, (**b**) miniprotein 63, and (**c**) miniprotein 76. The phosphorylation level of MEK1 was detected at varying concentrations of MBP and miniprotein inhibitors.

To further validate the biochemical potencies of miniproteins 63 and 76, in vitro kinase inhibition assays were conducted with respect to the phosphorylation of MEK1 by V600E mutant BRAF^15mut^. Figure 6 illustrates the phosphorylation levels of MEK1 in the absence and in the presence of miniprotein inhibitors at varying concentrations. No phosphorylated MEK1 signal is observed in the absence of V600E mutant BRAF^15mut^, implying that the auto-phosphorylation would be prohibited effectively by substituting Met for Lys at residue 97. However, the phosphorylated MEK1 signals appear explicitly upon the addition of V600E mutant BRAF^15mut^ due to the manifestation of kinase activity for MEK1. This indicates the establishment of a proper in vitro kinase assay system for evaluating the miniproteins. The phosphorylation signals of MEK1 also decrease as the concentrations of miniproteins 63 and 76 increase from 1.6 to 6 and 24 μM. The approximate IC_50_ values of miniproteins 63 and 76 associated with the inhibition of kinase activity amount to 8.0 and 1.0 μM, respectively. In contrast, the phosphorylation signal of MEK1 remains almost intact even when MBP exists at the high concentration of 24 μM in the absence of a miniprotein inhibitor. These results confirm that the decreases in the phosphorylation signal of MEK1 stem from the inhibitory activity of miniproteins 63 and 76 against V600E mutant BRAF^15mut^.

When the phosphorylated MEK1 signals derived from in vitro kinase assays of miniproteins 63 and 76 are compared (Figure 6), it can be deduced that the inhibitory activity of the latter is higher than that of the former. This differs from the results of BRAF binding assays in which the binding affinities of the two miniproteins with respect to V600E mutant BRAF^15mut^ were found to be similar (Figure 5). Such a similarity in the binding affinities may be understood in the context that both miniproteins reside at the bottom of the well under the ELISA conditions. However, the two miniprotein inhibitors can move freely under the conditions of the kinase inhibition assay in aqueous solution, which may enable miniprotein 76 to occupy a better position to bind to V600E mutant BRAF^15mut^ than miniprotein 63.

### 2.6. Cell-Based Assays

**Figure 7:**
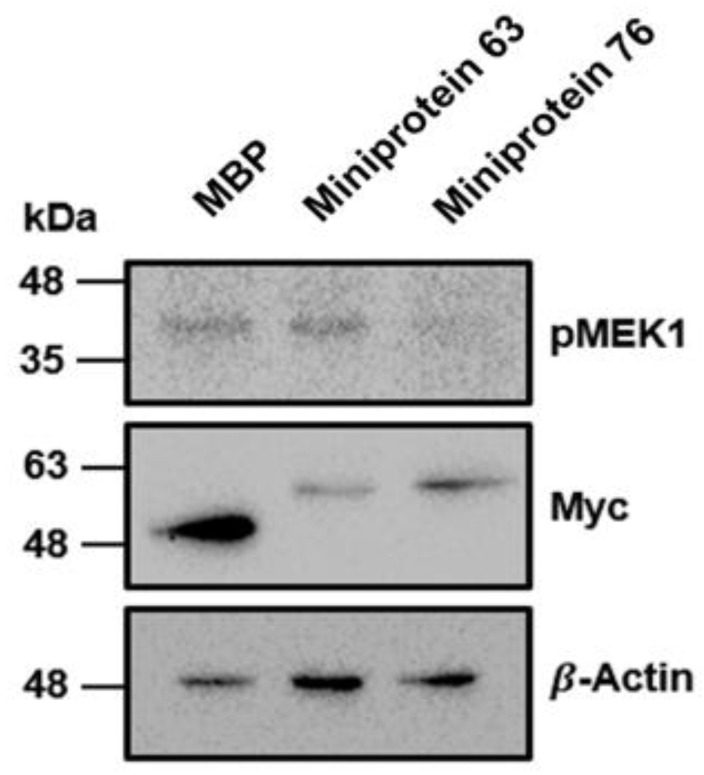
Western blots for the lysates of the three HeLa cells transfected with DNA encoding MBP, miniprotein 63, and miniprotein 76 separately. Myc bands are also presented to compare the expression levels of MBP and the two miniproteins, all of which contained the Myc-tag. The β-actin bands indicate that the amount of cell lysates is almost equal among the samples.

The biochemical potencies of miniprotein inhibitors were further assessed by examining the effects of intracellular expressions of 63 and 76 on MEK1 phosphorylation. HeLa cells involving the activated BRAF/MEK/ERK pathway [28,29] were used in these cellular assays because they might serve as an effective surrogate for cancer cells with hyperactive V600E mutant BRAF. Figure 7 shows the phosphorylation levels of MEK1 in the HeLa cells in which miniproteins 63 and 76 were transfected separately. HeLa cells with a transfected MBP gene were used as a control for the effects of transfection, protein overexpression, and MBP tag protein. Remarkably, the phosphorylated MEK1 signal becomes almost invisible in the presence of miniprotein 76 while the effect of miniprotein 63 appears to be as insignificant as that of MBP. Related with the difference in the inhibitory activities at the cellular level, it is interesting to note that the calculated solvation free energy of 76 is 2.9 kcal/mol lower than that of 63 (Figure 2b). This is in contrast to the relatively insignificant difference (0.4 kcal/mol) in binding energy scores (Figure 2a). Hence, the increase in the cellular biochemical potency in moving from miniprotein 63 to 76 may be attributed to the relatively high structural stability in an aqueous environment rather than the strengthening of binding to BRAF. Since significant biochemical potencies were demonstrated both in kinase inhibition assays and in cellular assays, miniprotein 76 is anticipated to serve as a good starting point to develop a new anticancer medicine against V600E mutant BRAF.

**Figure 8:**
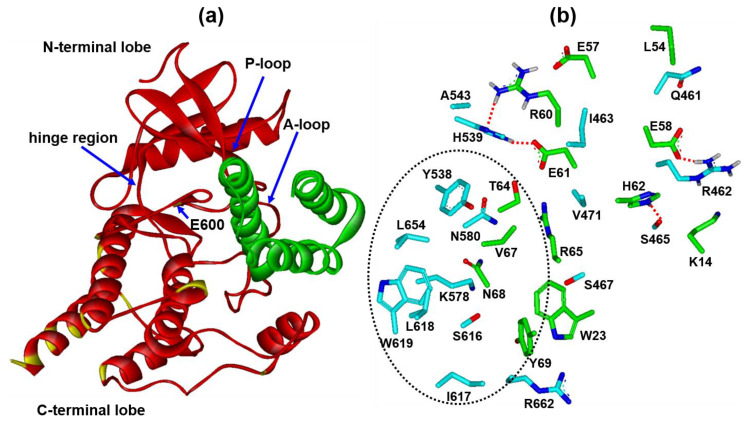
(**a**) Most probable docking pose of miniprotein 76 (green) with respect to V600E mutant BRAF^15mut^ (red). Colored in yellow are the residues mutated for aqueous solubility. (**b**) Amino-acid residues of BRAF (cyan) and miniprotein 76 (green) at the interface of the protein–protein complex. Red dotted lines and black dotted circles indicate the hydrogen bonds and van der Waals contacts, respectively.

We next turn to addressing the structural features relevant to the inhibitory activity of miniprotein 76 against V600E mutant BRAF. Figure 8 illustrates the most probable binding configuration of miniprotein 76 with respect to V600E mutant BRAF^15mut^, which was derived from docking simulations. The binding mode of miniprotein 76 with respect to BRAF differs from that of 14-3-3 in that the former is complexed between N- and C-terminal lobe of the kinase domain instead of the C-terminal tail for the latter (Figure 1a). In the calculated structure of the protein–protein complex, miniprotein 76 resides far from all 15 amino-acid residues mutated to enhance the aqueous solubility. It instead appears to interact with the phosphate-binding loop (P-loop, residues 462–469) and the activation loop (A-loop, residues 593–623) of V600E mutant BRAF^15mut^, both of which are the key structural elements for kinase activity. Judging from this binding mode, miniprotein 76 seems to prevent the phosphorylation of MEK1 by prohibiting the binding of both ATP and MEK1 to the kinase domain of BRAF.

With respect to the binding configuration derived with docking simulations between V600E mutant BRAF^15mut^ and miniprotein 76, it is noteworthy that Glu58 and His62 of the latter receive and donate a hydrogen bond from the sidechain guanidium ion of Arg462 and to the sidechain hydroxyl moiety of Ser465 at the P-loop of the former (Figure 8b), respectively. These two intermolecular hydrogen bonds would have the effect of preventing ATP from binding to the kinase domain of BRAF, culminating in the decrease in kinase activity [30]. In the calculated structure of the V600E mutant BRAF^15mut^-76 complex, two additional intermolecular hydrogen bonds are observed in which the sidechains of Arg60 and Glu61 of the latter act as the donor and acceptor with respect to the sidechain imidazole moiety of His539 of the former, respectively. These two hydrogen bonds are also likely to contribute to impeding the kinase activity of BRAF because His539 resides near the hinge region of the N- and C-terminal lobe [30]. Miniprotein 76 appears to be further stabilized upon complexation with V600E mutant BRAF^15mut^ through the van der Waals contacts of Thr64, Val67, Asn68, and Tyr69 with the sidechains of Tyr538, Lys578, Ser616, Ile617, Leu618, Trp619, and Leu654. Taken together, the low-micromolar inhibitory activity of miniprotein 76 against V600E mutant BRAF^15mut^ can be attributed to the combined effects of the multiple hydrogen bonds and hydrophobic interactions.

To enhance the biochemical potency of miniprotein 76 via structural derivatizations, it is noted that the sidechain of Asn68 points toward a small hydrophobic pocket comprising the sidechains of Tyr538, Leu618, Trp619, and Leu654 of BRAF. Therefore, the replacement of the hydrophilic Asn68 of 76 with a hydrophobic residue such as Phe or Trp would increase the inhibitory activity by strengthening the van der Waals contact with the target protein. The substitution of a nonpolar residue for Thr64 is also likely to increase the biochemical potency of 76 by establishing hydrophobic contacts with Tyr538. This induced hydrophobic interaction seems to be effective especially due to the proximity to the hydrogen bonds involving His539 (Figure 8b). Actually, the hydrophobic interactions tend to reinforce the vicinal hydrogen bonds by restricting the approach of hydrolytic water molecules. This protective role would become substantial because the neighboring hydrogen bonds involve the highly soluble ionic sidechains of Arg60 and Glu61 of 76. In this regard, the cooperative strengthening of hydrophobic interactions and hydrogen bonds may be a facile strategy for achieving the high biochemical potency of drug candidates [31,32]. Synergistic effects are therefore expected in strengthening the interactions between V600E mutant BRAF and miniprotein 76 by positioning the hydrophobic interactions and the hydrogen bonds in the same vicinity.

The binding mode of miniprotein 76 differs from those of small-molecule inhibitors in that the former binds extensively from the P-loop to the A-loop of BRAF instead of the accommodation in the small ATP-binding pocket. The lack of direct interactions with the amino-acid residues in the ATP-binding pocket is meritorious in terms of overcoming the resistance to BRAF inhibitors [33]. The structural modifications of miniprotein 76 can thus be made so as only to reinforce the binding to the target protein without considering the possibility of causing the manifestation of problematic drug-resistant mutants. Due to the low-micromolar inhibitory activity and good aqueous solubility, miniprotein 76 is anticipated to serve as a good starting point to develop new anticancer medicine for human cancers caused by V600E mutant BRAF. Future design of miniprotein inhibitors will focus on the enhancement of biochemical potency by augmenting hydrophobic interactions with nonpolar residues of the target protein.

## 3. Materials and Methods

### 3.1. Preparation of All-Atom Receptor Model for V600E Mutant BRAF

The 3D atomic coordinates of BRAF required for the de novo design of miniprotein binders were prepared from the X-ray crystal structure of BRAF (PDB entry: 4MNE) in complex with MEK1 [34]. Using the structure of the kinase domain (residues 449–722) of the wild-type BRAF, the construction of the all-atom model for V600E mutant BRAF began by inserting residues 465–468 that were missing in the original X-ray structure. The next step involved the introduction of 15 solubilizing mutations (I543A, I544S, I551K, Q562R, L588N, K630S, Y673S, A688R, L706S, Q709R, S713E, L716E, S720E, P722S, and K723G), which were necessary to ensure consistency with the experimental conditions for evaluating the candidate miniprotein inhibitors [25,26]. Finally, Val at residue 600 was replaced with Glu to complete the receptor model including a total of 16 mutations (V600E mutant BRAF^15mut^). The 3D atomic coordinates of V600E mutant BRAF^15mut^ were optimized with homology modeling using the latest version of the MODELLER program [35]. The original X-ray structure of the wild-type BRAF served as the structural template, from which different structures of V600E mutant BRAF^15mut^ were produced by conducting energy minimizations using the conjugate gradient algorithm and molecular dynamics simulations. This process aimed to reduce violations of spatial restraints. Among the ten structural candidates generated, we chose the one with the lowest MODELLER objective function value as the ultimate receptor model for V600E mutant BRAF^15mut^.

### 3.2. Preparation of the Miniprotein Scaffold for De Novo Design

As a starting structure for designing the miniprotein binders of V600E mutant BRAF, we used the substructure of the endogenous ligand protein (14-3-3), the binding of which to the C-terminal tail of BRAF induced the dimerization of BRAF [20]. Among 232 amino-acid residues of 14-3-3, those of the three α helices (residues 164–232) were selected as the structural scaffold of miniprotein inhibitors because they played a key role in binding to BRAF by encompassing the C-terminal tail [36]. In preparing the miniprotein scaffold from the three alpha helices of 14-3-3, we focused on determining the protonation states of ionizable residues, paying close attention to specific criteria. For instance, we considered the side chains of Asp and Glu residues as neutral if either of their carboxylate oxygens was within 3.5 Å of a hydrogen-bond-accepting group. Likewise, Lys residues were assumed to be protonated unless the NZ atom was close to a hydrogen-bond-donating group. We followed the same procedure for determining the protonation states of His residues. Finally, the AMBER program of version 12 [37] was used to add hydrogen atoms to heavy atoms, completing the all-atom model for the miniprotein scaffold.

### 3.3. Docking Simulations to Find the Most Probable Binding Configuration

Docking simulations between the V600E mutant BRAF^15mut^ and the miniprotein scaffold were carried out with the multi-scale Monte-Carlo-based algorithm as implemented in the RosettaDock program [38]. The initial binding configuration was prepared by superimposing the miniprotein scaffold on MEK1 in the original structure BRAF-MEK1 complex. The miniprotein was then translated and rotated to find the optimal configuration with respect to the fixed structure of V600E mutant BRAF^15mut^. More specifically, the most probable binding modes were searched so as to optimize the sidechain positions of the miniprotein via rotamer packing and explicit gradient-based minimization of the rigid-body displacements. The binding energy function to score the putative receptor–ligand complexes consisted of van der Waals energies, low-weighted electrostatics energy, implicit Gaussian solvation, orientation-dependent hydrogen bonding, and side-chain rotamer probabilities [39]. Of 1000 binding configurations generated during the docking simulations, the one with the lowest binding energy was selected as the final structural model for V600E mutant BRAF^15mut^ in complex with the miniprotein scaffold.

### 3.4. De Novo Design of Miniprotein Inhibitors of V600E Mutant BRAF

Starting from the most probable binding configuration, all 69 amino-acid residues of the miniprotein scaffold were allowed to mutate to find the structurally stable miniproteins that could bind tightly to V600E mutant RBAF^15mut^. All these putative miniprotein binders were prepared with the RosettaRemodel blueprint format [40] under the constraint that the tertiary structure should maintain the three alpha helices bundle as in the starting structure. Preliminarily, the homology-modeled structure of V600E mutant BRAF^15mut^ was loaded into a hashing grid to check the presence of bad van der Waals contacts at the interface of the receptor–ligand complex. The entire structures of miniproteins, including the key interacting residues, were then designed with the FastDesign protocol [41] that implemented the optimization of side-chain rotamers and energy minimizations using the gradient-descent method. The amino-acid residues at the binding interface were further optimized to reflect the shape complementarity between the receptor and a ligand. Actually, all 69 amino acids were allowed to mutate to produce new miniprotein ligands, which were scored according to the quality of the predicted 3D structure, as well as to the strength of the interactions with V600E mutant BRAF^15mut^. Among a total of 1000 miniproteins generated with the ROSETTA scoring function, 100 top-scored miniproteins were selected for further analysis.

### 3.5. Rescoring the Putative Miniprotein Binders with the Hydration Free Energy

To calculate the hydration free energy (Δ*G_sol_*) of each miniprotein inhibitor candidate, the solvent-contact model was adopted under the assumption that Δ*G_sol_* of a miniprotein would be given by the sum of atomic contributions.

(1) $$\Delta G_{sol}=\sum_{i}^{atoms}\Delta G_{sol}^{i}$$

The atomic hydration energy of atom *i* was approximated by multiplying the atomic volume exposure to bulk solvent (*F_i_*) and the atomic hydration parameter (*S_i_*) as follows.

(2) $$\Delta G_{sol}^{i}=S_{i}F_{i}$$

*F_i_* can be calculated by subtracting the occupied volume of the atom *i* (*O_i_*) from the maximum atomic volume (*O_i_^max^*). In a physical sense, *O_i_* refers to the volume to which the approach of a solvent molecule is restricted due to steric hindrance by the rest of the miniprotein atoms. The *O_i_* parameter of atom *i* is usually obtained by summing the product of the atomic fragmental volume parameters (*V_j_*’s) of all the other atoms and the Gaussian-type envelope function with respect to the distance between atoms *i* and *j* (*r_ij_*) [42]. Finally, Δ*G_sol_* of a miniprotein can be expressed as follows.

(3) $$\Delta G_{sol}=\sum_{i}^{atoms}S_{i}\left(O_{i}^{\max}-\sum_{j}^{i\neq j}V_{j}e^{-\frac{r_{ij}^{2}}{2\sigma^{2}}}\right)$$

The atomic *S_i_*, *O_i_^max^*, and *V_j_* parameters were extracted from those optimized with the standard genetic algorithm using the hydration free energy data of dipeptides [26]. The *σ* parameter in the Gaussian-type envelope function was set equal to 3.5 Å to define the atomic volume to which the access of solvent molecules is forbidden. In computing the hydration energy of a potential miniprotein binder using Equation (3), we adopted the *S_i_*, *O_i_^max^*, and *V_i_* parameters as detailed by Chung and Park [43], known for their effectiveness across diverse organic compounds. Including this advanced hydration energy term in the scoring function was expected to enhance the accuracy of de novo design by accounting for ligand hydration effects in BRAF–miniprotein binding. Final candidate miniprotein inhibitors for experimental validations were thus selected using the hydration energy calculated with Equation (3).

### 3.6. Molecular Constructs

DNA encoding the BRAF kinase domain (residues 444–723; UniProt ID: P15056) with 15 solubilizing mutations and V600E mutation was synthesized at Bioneer Corp. (Daejeon, Republic of Korea). The synthesized construct of V600E mutant BRAF^15mut^ was cloned into a pET-32a vector along with the tag of N-terminal maltose-binding protein (MBP) using the restriction enzymes NdeI and XhoI (New England Biolabs, Ipswich, MA, USA). DNA encoding MEK1 (residues 1–393; UniProt ID: Q02750) was ordered from Addgene (Watertown, MA, USA). This construct was subcloned into pET-32a vector at the same restriction sites as V600E mutant BRAF^15mut^ with N-terminal MBP-tag, followed by the insertion of C-terminal His-tag via a polymerase chain reaction. In the case of MEK1, the kinase-dead mutation (K97M) was introduced using the QuikChange site-directed mutagenesis protocol (STRATAGENE, La Jolla, CA, USA) to prevent problematic auto-phosphorylation. C-terminal Myc tag was also appended to K97M mutant MEK1 to facilitate the BRAF binding assays. All DNA sequences were confirmed via Sanger DNA sequencing.

Plasmids of 39 candidate miniprotein inhibitors possessing the C-terminal Myc tag were synthesized and subcloned into a pMAL-c5x vector using NdeI and EcoRI sites at Synbio Technologies (Monmouth Junction, NJ, USA). In particular, the two miniproteins screened from BRAF binding assays and kinase inhibition assays were subcloned with MBP into pcDNA™ 3.1/myc-His A vector using the KpnI and XhoI restriction enzymes (New England Biolabs, Ipswich, MA, USA) to be transfected in HeLa cells.

### 3.7. Expression and Purification of Proteins

All proteins were expressed with *E. coli* strain BL21(DE3). After growing the cells in Luria–Bertani (LB) medium supplemented with 100 μg/mL of ampicillin until the OD600 value reached 0.6–0.8, proteins were induced using isopropyl β-D-1-thiogalactopyranoside (IPTG) at the concentration of 1 mM. The bacterial cells induced at 18 °C were spun down by centrifugation at 6000 rpm for 10 min and stored at −80 °C. All protein purifications were performed at 4 °C and began by lysing the harvested cells using a Digital Sonifier^®^ (Branson, Danbury, CT, USA) in lysis buffer (50 mM Tris-HCl pH 7.5, 500 mM NaCl, 5 mM β-mercaptoethanol) at 65% amplitude. The lysate was then centrifuged at 16,500 rpm to filtrate the supernatants, which were incubated with a prepacked MBPTrap^®^ HP column (Cytiva, Marlborough, MA, USA). The incubated proteins were then washed and eluted with elution buffer (50 mM tris-HCl pH 7.5, 200 mM NaCl, 3 mM maltose, 5 mM β-mercaptoethanol).

MEK1 was incubated with Ni-NTA resin (Qiagen, Hilden, Germany) using the same equilibrium buffer as in the MBPTrap^®^ HP column (50 mM Tris-HCl pH 7.5, 200 mM NaCl, 5 mM β-mercaptoethanol). The resin was washed in two steps with the first wash buffer (50 mM Tris-HCl pH 7.5, 1 M NaCl) and the second wash buffer (50 mM tris-HCl pH 7.5, 200 mM NaCl, 30 mM imidazole, 5 mM β-mercaptoethanol) and then eluted with the elution buffer (50 mM Tris-HCl pH 7.5, 200 mM NaCl, 0.5 M imidazole, 5 mM β-mercaptoethanol). After concentrating the fractions with Amicon^®^ Ultra-15 Centrifugal Filter (10 kDa cutoff, Merck, Darmstadt, Germany), the protein was desalted with TBS (20 mM Tris-HCl pH 7.5, 150 mM NaCl, 5 mM β-mercaptoethanol) using a HiTrap^®^ Desalting column (Cytiva, Marlborough, MA, USA) to remove the remaining imidazole in the solution.

### 3.8. BRAF Binding Assays

In the first step for validating the biochemical potencies of the designed miniproteins via enzyme-linked immunosorbent assay (ELISA), 10 μg/mL of V600E mutant BRAF^15mut^ was coated in a 96-well plate. Each coated well was washed with TBS-T (20 mM Tris-HCl pH 7.5, 150 mM NaCl, 0.05% Tween^®^ 20) and blocked with the blocking buffer (20 mM Tris-HCl pH 7.5, 150 mM NaCl, and 5% (*w*/*v*) skim milk). Subsequently, the purified Myc-tagged proteins including MEK1, MBP, and miniproteins were mixed in each well at varying concentrations of miniproteins (0, 200, and 2000 nM). After the incubation, an anti-c-Myc antibody (1:500 dilution) purchased from Santa Cruz Biotechnology (#sc-40, Santa Cruz, CA, USA) was added, followed by the addition of horseradish peroxidase (HRP)-conjugated goat anti-mouse IgG antibody (1:5000 dilution) and further incubation for 2 h. After colorizing each well with the TMB solution, 50 μL of stop solution was added to stop the binding reactions. The absorbance of each well was measured at the wavelength of 450 nm using EMax Microplate Reader (Molecular Devices, Sunnvale, CA, USA). All absorbance measurements were conducted in triplicate at varying concentrations to observe the dose–response behaviors of absorbance. These triplicate measurements were duplicated for MEK1, MBP, miniproteins 63 and 76 to confirm the consistency in the main results.

### 3.9. In Vitro Kinase Inhibition Assays

V600E mutant BRAF^15mut^, MEK1, and candidate miniprotein inhibitors were mixed to initiate the enzymatic reactions. Single concentrations of V600E mutant BRAF^15mut^ and MEK1 were set to 0.2 and 1 μM, respectively, while each miniprotein was added at varying concentrations of 1.5, 6, and 24 μM. After inducing the phosphorylation of MEK1 by V600E mutant BRAF^15mut^ in the reaction buffer (50 mM Tris-HCl pH 7.5, 10 mM MgCl2, 10 mM DTT, and 1 mM ATP), the reaction was stopped by adding 5X SDS-PAGE sample loading buffer. Each reaction mixture was then separated by loading on 10% SDS-polyacrylamide gel. All the separated proteins were transferred to PVDF membrane activated by methanol using the Trans-Blot^®^ SD semi-dry transfer cell (Bio-Rad, Hercules, CA, USA). After blocking the membrane with 5% BSA in TBS-T (20 mM tris-HCl pH 7.5, 150 mM NaCl, 0.1% Tween^®^ 20), the membrane was incubated with rabbit anti-p-MEK1 antibody against Ser217/Ser221 (1:1000 dilution) in 5% BSA with TBS-T. The next step involved washing with TBS-T and further incubation of the membrane with HRP-conjugated goat anti-rabbit IgG antibody (1:500 dilution) in 5% skim milk with TBS-T. After adding Pierce™ ECL Western Blotting Substrate (ThermoFisher, Waltham, MA, USA) to the membrane, chemiluminescent signals were detected via the ChemiDoc XRS+ System (Bio-Rad, Hercules, CA, USA) to visualize the protein bands. The IC_50_ values of miniproteins 63 and 76 were measured in duplicate by monitoring the changes in band intensities.

### 3.10. Cell Culture and DNA Transfections

Human cervical cancer HeLa cell lines were used to validate the cellular-level efficacy of the two miniprotein inhibitors identified in the kinase assays. HeLa cells were grown in Dulbecco’s Modified Eagle’s Medium (Welgene, Kyeongsan, Republic of Korea) containing 10% FBS and 1% antibiotic-antimycotic, then incubated with 5% CO_2_ at 37 °C. The transfection of DNA encoding the miniprotein inhibitors with N-terminal MBP tag was carried out using Lipofectamine™ 3000 Transfection Reagent (Invitrogen, Carlsbad, CA, USA). The construct encoding MBP was also transfected in HeLa cells separately to investigate the effect of the presence of MBP tag on the reduction in the phosphorylation signal of MEK1. The expression of each recombinant protein was detected via Western blot as detailed below.

After the transfections and the removal of culture medium, cells were lysed with modified RIPA buffer (25 mM tris-HCl pH 7.6, 150 mM NaCl, 1% NP-40, 1% sodium deoxycholate, 0.1% SDS, 1X protease inhibitor cocktail, and 1X Xpert phosphatase inhibitor cocktail solution). The lysate was centrifuged to collect the supernatant containing the proteins, the concentrations of which were determined using a DC Protein Assay Kit (Bio-Rad, Hercules, CA, USA). Subsequently, 20 μg of each protein was separated via SDS-PAGE in which blocking, primary and secondary antibody binding, and band visualizations were carried out in the same way as in the kinase inhibition assays. The anti-phospho-MEK1/2 (Ser217/221) antibody was used to detect the phosphorylated MEK1 (#9121, Cell Signaling Technology, Beverly, MA, USA). Because all proteins have a C-terminal Myc tag, the anti-Myc antibody was used for the determination of the protein expressions in HeLa cells. The Western blot experiments in cell-based assays were carried out also in duplicate to ensure the miniprotein-dependent pMEK1 profiles.

## 4. Conclusions

Starting from the substructure of the endogenous ligand protein (14-3-3), a structure-based de novo design was carried out to identify the miniprotein inhibitors of V600E mutant BRAF. Tentatively, 39 miniproteins comprising three α-helices and 69 amino acids were selected as candidate inhibitors through a two-step scoring scheme involving the binding affinity to the target protein and the hydration free energy. As a consequence of serial biochemical evaluations with in vitro binding assays and kinase inhibition assays, two miniproteins (63 and 76) were identified as new inhibitors of V600E mutant BRAF15mut with low-micromolar activity. In particular, miniprotein 76 impeded the phosphorylation of MEK1 explicitly in mammalian cells, implying that it could serve as a good starting point to develop new therapeutics for cancers caused by BRAF. The docking simulation results indicate that miniprotein 76 can bind tightly to BRAF through the four hydrogen bonds with the sidechains on the P-loop and at the top of the C-lobe, along with the van der Waals contacts with the nonpolar residues on the A-loop.

## Figures and Tables

**Table 1:** Amino acid sequences of 24 candidate miniproteins purified in soluble form.

No	Amino Acid Sequence
21	AVIKNKALQMCWNRTCVWAANEQLQASLDLIVFYMSGLRNTCLMLMLRLMFVSELLVLDRELHKDMVAV
33	APKREVAFMELLTRCLVTVLNNQAQTKLQLIDCYLADLLMRVLNRLAMMMLMAHLNLLMQLAYQGMHAQ
45	ADIMMCALGWEISVVKVFGYNNPSMDQLLNNKWFLGNGWNYKFMRMEVMLGMRAWSLTTVENLLIELIG
51	LVSCQFNSMWKFTSMEVWNYNTMLASQMWGHVYTNATLLANLLMGMMIMCKKLRLTSWMATDVVLGGDW
59	GHRCNMARANLLTSMASIGLYTMYDGIMYTECILIHDNQIRVKMWLAQMFKMEVSAFDLRDDSSLYHLG
60	QANGMTTLMFLNILNWVWHYQTMPRAAEDGYVHWLNIGVKMELMRMCWCMWKARACAHMLQDDTQFSSH
63	SIVGQGAQQMLVSKIKCTVANEWLDRSMLTSKFAMFVLLNEIDMLDIMLEAMGISLLEDREHQTRRVNY
64	PDKCAMASEWLLGTNWCWMYYGRQASIYQTIHFYLAMKAGYQFVYMFVMMAMRHWLIAMMRKDYRMLAK
70	AVINQVAWDQWNTLMTPDVYACCLIALRLGIFTALACGTARLDMRYTIRQEDKQLVMQMNLLDKIVFEV
71	AVIRDVAKWWLLHNMWSIVQTNQQIGNTHDQVCNSYDTLVRVLMSPEMIFDATRLDNFEVLHCYLMMQV
75	CNSLILKWLLFGGDHWHLRGVTQILTREIACTAWMEQWLSVDSSLMSNNRASNYMNVERCWLWKETDAA
76	SIQGQGARQCLVSKLKCTVANEWLDRWMLTSKFAMFVLLNEIDMLDIMLEAMGISIEEDREHQTRRVNY
77	GTIDQRWWNHFDSTTERTVHEMTSDCHMRMQKSMTQNRCSMVVVPHRMRYVGYKGMRNINNMGYCGDAA
78	LELLRAEETAHTSTLHIDVEVISTTAFAYCRMDQFTQISNDALETMNQLESGLAWMAVRGCYMMTCVGE
79	VTMQFSFNSLLEGRAEAHMGTILITAMAITIKKLKGRGAFGKKLLEWNMAMACSHNAQLGGMDFQHKEG
83	SEGAAKASELLQSMNLCWMYIGCQNSIQQTIAVYLAMQAGMQFVYMFVCSAAQHWLKTMDRKNYLMLVK
84	ADDQNTEIVWLLVSQWWWLYNFVEIANLLLVSNMNTIWNNRMKGRMNMNCLMYRMLGNRGMLCLQDGQA
86	TAVLIVFLEGLSAATGRHVGARMPDAAYSKNYHMMFAAAQACDYRGNMKSLARGFAAAQWMMTWVSFIS
91	NACEFKALDVDRGNLHDAHRNCGGRKNNAMQVEAMIVLSNGFGAGPNVWMLKAALLEVMTLLTCDTLKR
92	GVIRGHARVRALAGRMYALLDVTWCADSKSRRIMQGGFLVHIFNQEWTLYGELAKTTWVWRHFFQIMDF
93	CKQMQALHAFSSEADRQHLQACAWKGMNVGCVLEVVVLDAIMGNQNNDQYFSTNIMMQMSTVFWNLQGM
94	PCVWTGFREFKGGQRGKERLAMCEDLMCEADWWGGNQNRDRGSIFTRVKGSGALGKISIAERGARNNAV
98	TMNTIMMKVSFCAGARIVHLAVLVIGLDVDRSELMDTAIDTMVHWNFAISEVYMVLWGSSQSKSDCQEV
100	ACTETIWTVTCNGHLEMRTYWAYCAMFTFMESHITDNLHWTAAMFTQEVRTRTFYAGVQGNSMWWTVRN

## Data Availability

The data can be shared up on request.

## Supplementary Materials

The following supporting information can be downloaded at: https://www.mdpi.com/article/10.3390/ijms25105535/s1.
