# Supplementary material for: Structure-Based De Novo Design for the Discovery of Miniprotein Inhibitors Targeting Oncogenic Mutant BRAF
Source: Int J Mol Sci. 2024 May 19;25(10):5535. doi: 10.3390/ijms25105535 (PMC11122373; doi:10.3390/ijms25105535)

Supplementary Material

Structure-Based *De Novo* Design for the Discovery of Miniprotein Inhibitors Targeting Oncogenic Mutant BRAF

Jae Min Ham[a,1], Myeongbin Kim[a,1], Taeho Kim[2], Seong Eon Ryu[1,] and Hwangseo Park[2,*]*

[1]Department of Bioengineering, College of Engineering, Hanyang University, 222 Wangsimri-ro, Seong-dong-gu, Seoul 04763, Korea.

[2]Department of Bioscience and Biotechnology, Sejong University, 209 Neungdong-ro, Kwangjin-gu, Seoul 05006, Korea

[a]These authors contributed equally to this work.

Figure S1. Amino acid sequence alignments of 14-3-3 (residues 164-232) with miniproteins (a) 76 and (b) 63. Identical and similar residues are colored in red and green, respectively.

(a)

```
14-3-3   164 THPIRLGLALNFSVFYYEILNSPDKACQLAKQAFDDAIAELDTLNEDSYKDSTLIMQLLR
miniprot   1 SIQGQGARQCLVSKLKCTVANEWLDRWMLTSKFAMFVLLNEIDMLDIMLEAMGISIEEDR

14-3-3   224 DNLTLWTSD   232
miniprot 61 EHQTRRVNY    69
```

(b)

```
14-3-3   164 THPIRLGLALNFSVFYYEILNSPDKACQLAKQAFDDAIAELDTLNEDSYKDSTLIMQLLR
miniprot   1 -SIVGQGAQQMLVSKIKCTVANEWLDRSMLTSKFAMFVLLNEIDMLDIMLEAMGISLLED

14-3-3   224 DNLTLWTSD-   232
miniprot 60 REHQTRRVNY    69
```

Figure S2. 3D structural alignments of (a) backbone groups and (b) sidechains of 14-3-3, miniprotein 76, and miniprotein 63. Sidechain atoms of 14-3-3, miniprotein 76, and miniprotein 63 are indicated in green, cyan, and blue, respectively.

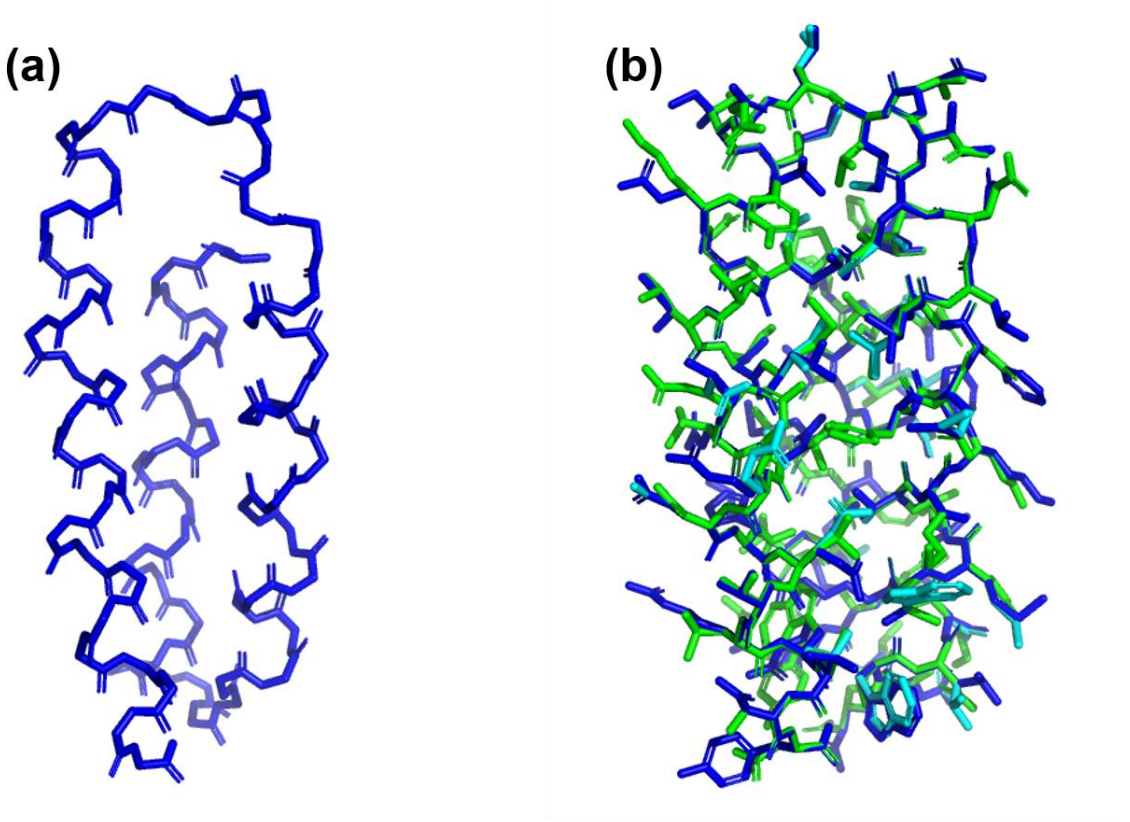

Supplement: Supplementary file 1 [file ijms-25-05535-s001.zip › ijms-2977447-supplementary.pdf]
